# Morphometric Parameters and MRI Morphological Changes of the Knee and Patella in Physically Active Adolescents

**DOI:** 10.3390/medicina59020213

**Published:** 2023-01-22

**Authors:** Goran Djuricic, Filip Milanovic, Sinisa Ducic, Vladimir Radlović, Mikan Lazovic, Ivan Soldatovic, Dejan Nikolic

**Affiliations:** 1 Faculty of Medicine, University of Belgrade, 11000 Belgrade, Serbia; 2 Radiology Department, University Children’s Hospital, 11000 Belgrade, Serbia; 3 Orthopedic Surgery and Traumatology Department, University Children’s Hospital, 11000 Belgrade, Serbia; 4 Physical Medicine and Rehabilitation Department, University Children’s Hospital, 11000 Belgrade, Serbia

**Keywords:** knee injury, magnetic resonance imaging, children, physical activity, overuse syndrome

## Abstract

*Background and Objectives*: The immature skeleton in a pediatric population exposed to frequent physical activity might be extremely prone to injuries, with possible consequences later in adulthood. The main aim of this study is to present specific morphometric parameters and magnetic resonance imaging (MRI) morphological changes of the knee and patella in a physically active pediatric population. Additionally, we wanted to investigate the morphological risk factors for patellar instability. *Materials and Methods:* The study included the MRI findings of 193 physically active pediatric patients with knee pain. The participants underwent sports activities for 5 to 8 h per week. Two divisions were performed: by age and by patellar type. We evaluated three age groups: group 1 (age 11–14), group 2 (age 15–17), and group 3 (age 18–21 years). In addition, participants were divided by the patellar type (according to Wiberg) into three groups. The following morphometric parameters were calculated: lateral trochlear inclination (LTI), the tibial tubercle–trochlear groove distance (TT-TG), trochlear facet asymmetry (TFA), Insall–Salvati index, modified Insall–Salvati index, Caton–Deschamps index, articular overlap, morphology ratio and contact surface ratio. *Results:* We found a statistically significant association between patellar type groups in LTI (*p* < 0.001), TFA (*p* < 0.001), Insal–Salvati (*p* = 0.001) index, and Caton–Deschamps index (*p* = 0.018). According to age groups, we found statistical significance in the Caton–Deschamps index (*p* = 0.039). The most frequent knee injury parameter, according to Wiberg, in physically active pediatric patients was patella type 2 in boys and type 3 in girls. *Conclusions:* The MRI morphometric parameters observed in our study might be factors of prediction of knee injury in physically active children. In addition, it might be very useful in sports programs to improve the biomechanics of the knee in order to reduce the injury rate in sports-active children.

## 1. Introduction

Nowadays, the availability of a wide range of sports and the need for participation result in more children being physically active, particularly in competitive sports. The negative effect of improperly adapted exercise on the immature skeleton is well described in the literature [1,2,3]. However, an awareness of the potential unfavorable consequences is lacking. As a result, the incidence of sports injuries in the pediatric population due to unsuitable physical activity is increasing [4]. Repeated exercises might lead to overuse injuries of the overloaded joints and muscles due to the disproportion between musculoskeletal structures [5]. Physically active children are often referred to a doctor because of recurrent pain after excessive activity [6,7]. The most common consequence of those nonspecific, and at first glance insignificant, symptoms is an oversight of the hidden etiopathology [8]. The recurrence of such pain is rarely followed by any abnormal clinical and radiological findings, meaning patients might not be diagnosed in a timely fashion or might be inadequately treated.

Magnetic resonance imaging (MRI) is considered the reference standard modality in the radiological diagnosis of joints and soft tissue lesions. The high resolution offers excellent multiplanar imaging and differentiation of soft tissue, bone marrow, cartilage, muscles, ligaments, and tendons. Moreover, the absence of the negative effects of ionizing radiation makes MRI the method of choice to evaluate sports injuries in the pediatric population [9].

An analysis of the morphometric parameters of the patellofemoral joint and an assessment of their deviations from ideal values in the presence of patellofemoral dysplasia, as well as their relationship with the presence of risk factors for more frequent and extensive knee injuries in physically active children, were complemented by analyzing the patella type according to Wiberg, both for males and for females. Contrary to previous published works where [10,11] separately investigated these two risk factors, here we examined their joint impact and the mutual relationship between them. This is of particular importance both in the diagnosis and in the screening of sports-active children, with the aim of preventing injuries with a timely physiotherapy and conditioning program to improve knee biomechanics in people who are at the beginning of their sports career.

The main aim of this study is to present specific morphometric parameters and MRI morphological changes of the knee and patella in a physically active pediatric population. Additionally, we aimed to investigate the morphological risk factors for patellar instability. 

## 2. Patients and Methods 

In this prospective study, we observed the MRI findings of 193 physically active pediatric patients treated at the University Children’s Hospital who reported pain in the knee joint during sports activities. Informed consent for participation was obtained from all subjects involved in the study. 

Inclusion criteria were as follows: pediatric patients over 11 years old who participated for more than five and less than eight hours of sports activities per week during a period of more than three years of active training, followed by clinical examination and knee radiography in two views (AP and lateral view), without any pathological clinical and X-ray findings. Patients with osteomyelitis, bone tumors, juvenile rheumatoid arthritis, and metabolic diseases were excluded, as well as patients with previously treated trauma of the knee. The study was approved by the Institutional Review Board and followed the principles of good clinical practice (protocol number 017-16/15, 9 April 2021).

Two divisions of our study sample were performed: the first one considered the age of participants, and the second considered the MRI type of patella, according to Wiberg patellar classification. In the Wiberg classification, patellar facet sizes are classified into three types based on their relative medial and lateral sizes. The Wiberg classification initially applied to radiography but is now used in other modalities. [12]. Most patellae are Wiberg type 2, accounting for up to 65%. Type 3 accounts for 25%, and type 1 accounts for 10% [13]. In type 1 patella, the medial and lateral patellar facets are similar in size and are both concave; in type 2, the medial patellar facet is shorter than the lateral facet, and both facets are concave; in type 3 patella, the medial facet is much shorter than the lateral facet and convex [12]. Furthermore, a type 4 was described by Baumgartl, the “Jaegerhut” patella, with no medial facet and consequently no median ridge [12]. Age groups were set according to the American Academy of Pediatrics (AAP) classification; patients were divided into three age groups: group 1 (age 11–14 years), group 2 (age 15–17 years), and group 3 (age 18–21 years) [14]. MRI scans were performed on Siemens MagnetomAera 1.5T using a knee coil, positioned in supination with a field of view (FOV) of 16 cm using Ax Int FS, Cor Int FS, Cor T1, Sag Obl Int FS, Sag Obl PD, Cor Obl PD. The axial sections were parallel to the knee joint line; they included the entire patella and the head of the fibula. The coronal sections were parallel to the femoral condyle’s posterior aspect—including the entire patella and up to 2 cm posterior to the femoral condyle. The sagittal sections were parallel to the medial aspect of the lateral condyle, including both collateral ligaments.

Magnetic resonance imaging was processed using Syngo via software to mark the following morphometric parameters: lateral trochlear inclination (LTI), trochlear facet asymmetry (TFA), the trochlear depth, the tibial tubercle–trochlear groove (TT-TG) distance, articular overlap, morphology ratio, and contact surface ratio. 

**Figure 1:**
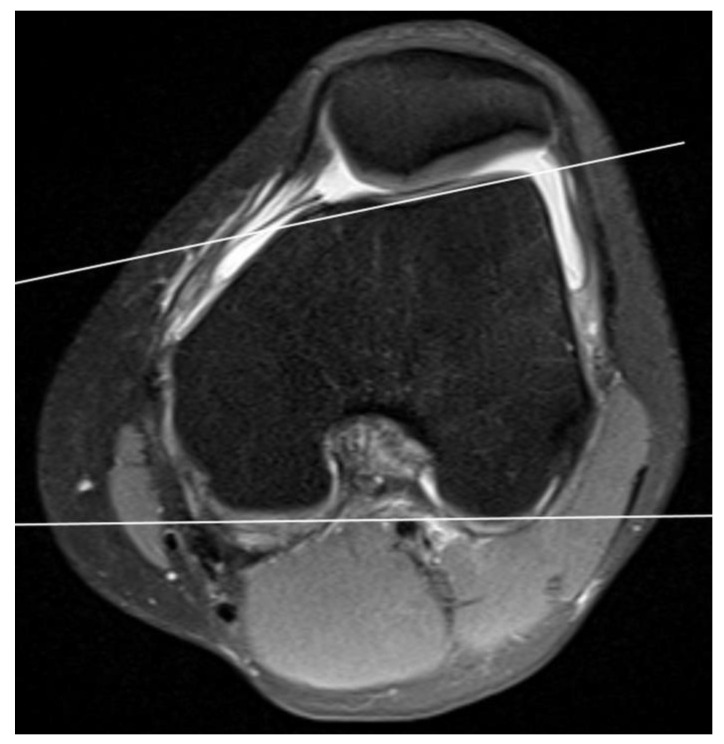
Proximal trochlear articular surface and measuring of lateral trochlear inclination.

The lateral trochlear inclination was determined from the first axial image in which cartilage was present and was used as a reference image. This angle corresponds to the angle between the lateral edge of the femoral epicondyles and the posterior edge of the femur. A line tangential to the subchondral bone of the posterior aspect of the two femoral condyles was crossed with a line tangential to the subchondral bone of the lateral trochlear facet to calculate LTI. A fat-suppressed PD-weighted image clearly delineates cartilage as a thick band of intermediate signal intensity adjacent to subchondral bone. When the inclination angle is less than 11°, trochlear dysplasia is diagnosed (sensitivity 93%; specificity 87%) [15] (Figure 1). 

**Figure 2:**
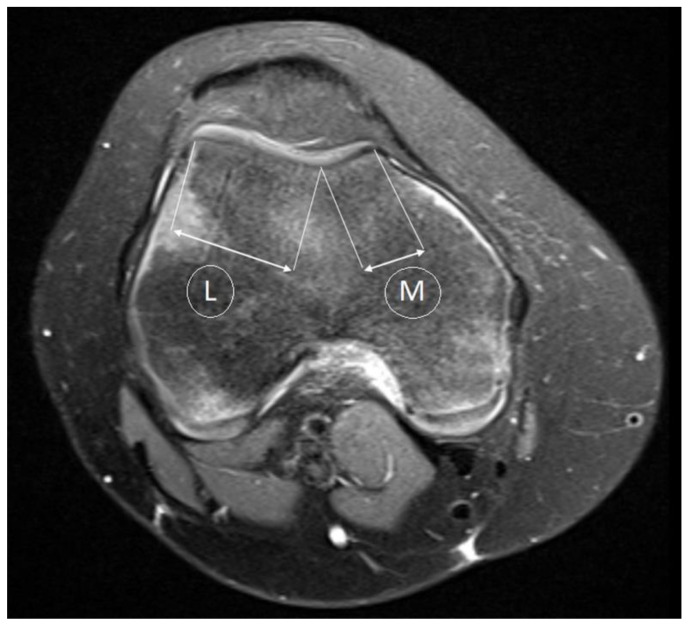
Trochlear facet asymmetry, ratio of length of medial trochlea to length of lateral trochlea. L—lateral, M—medial.

The morphology of the trochlear facet asymmetry assessed on axial fat-saturated PD-weighted MR images, from the first axial image in which the articular cartilage was present. In this image, the greatest length and articular thickness of cartilage is approximately 3 cm above the tibiofemoral joint cleft. The asymmetry of the trochlear facet was measured as the ratio of the medial to lateral faces. In order to calculate asymmetry in the length of the medial facet (M) and the lateral facet (L), the medial facet length was divided by the lateral facet length and expressed as a percentage (M/L × 100%). In the presence of dysplasia, a trochlear facet ratio below 40% was considered indicative (96% specificity; 100% sensitivity) [16] (Figure 2).

Using transverse sections, the trochlear depth was calculated using the formula ([A + B]/2 − C) given by Pfirmann et al. [17]. By measuring the maximum anteroposterior distance of the medial and lateral femoral condyles, distances A and B were determined. The distance C is the minimum anteroposterior distance between the deepest point of the trochlear groove and the line paralleling the posterior outlines of the femoral condyles.

Furthermore, the TT-TG measures the distance of the trochlear groove from the tibial tubercle. By using the posterior plane of the condyles as a reference line, the distance between the deepest point of the trochlea and the middle of the tibial tubercle was measured. In individuals with severe trochlear dysplasia, measuring the lateral distance between the tibial tubercle and the trochlear groove is less accurate because it is difficult to determine the depth of the trochlea. A distance of less than 15 mm between the tibial tubercle and the trochlear groove indicates normal tuberosity, a distance between 15 and 20 mm is borderline, and a distance longer than 20 mm indicates marked lateralization. It is nearly always associated with patellar instability if the distance between the tibial tubercle and trochlear groove exceeds 20 mm [16,18].

Additionally, we also observed patella alta ratios, and Insall–Salvati, modified Insall–Salvati, and Caton–Deschamps indices. Patella alta occurs when the patellar tendon is too long and rises too high above the trochlear fossa. Compared with a normal knee, patella alta requires a greater degree of flexion for the patella to engage in the trochlea, resulting in patellofemoral misalignment. In shallow degrees of flexion, this problem reduces the patellar contact area and decreases bone stability [19,20].

According to the Insall–Salvati ratio, patellar height is determined by the length of the patella tendon to the length of the patella. Sagital MR images are reliable tools for measuring patellar height ratios since they demonstrate the true anatomy of the patellofemoral joint and its ligaments in three dimensions. Distance lines for patellar tendon length (A), which represents the maximum length from the lower pole of the patella to its insertion on the tibia, and patellar length (B) are used in the calculation of Insall–Salvati ratio (A/B). An alternative method for assessing patellar height is the modified Insall–Salvati ratio. A proposal was made to avoid mismeasurements due to the variable shape of the inferior patella pole. The modified Insall–Salvati ratio (C/D) measures the distance from the inferior margin of the patellar articular surface (C), as opposed to the lower pole of the patella itself, to the patellar tendon insertion length of the patellar articular surface (D). Caton–Deschamps indices are based on the length of patellar articular surfaces and their distance from the tibia, which reduces erroneous measurements in patellas with long bodies, as measured by the Insall–Salvati ratio. The Caton–Deschamps index measures (A) the distance between the anterior angle of the tibial plateau and the most inferior aspect of the patellar articular surface length (B). Caton–Deschamps index = A/B. The Insall–Salvati ratio is considered normal for the values 0.8–1.2 (or values <2, according to MOD Insall–Salvati), whereas the Caton–Deschamps normal range is considered 0.6–1.3 [19].

The patellar trochlear overlap measures the articular overlap of the patellar undersurface and femoral trochlear cartilage (mm). The length of patellar cartilage (B) overlying the trochlear cartilage (A) was measured parallel to the subchondral surface of the patella using sagittal MRI. As an independent variable, the uncovered length of patellar cartilage (B) was also measured to calculate articular overlap as a percentage of overall articular length. Percentage coverage = (A/B) × 100 [21].

All measurements were performed independently by two observers, both with previous experience of more than 10 years in interpreting MRI findings, in order to evaluate interobserver reliability.

## 3. Statistical Analysis

The collected data were statistically analyzed using the IBM SPSS software package (IBM Corporation, New York NY, USA), version 21, using categorical and continuous variables. Descriptive statistics were used in order to determine the frequency of various pathological changes in knee joint injuries. A Pearson chi-square test, Fisher’s exact test, and chi-square test for trend were performed. A statistical significance was considered at the level of *p* < 0.05. Continuous variables with a normal distribution were described using mean value and standard deviation (SD) to calculate the age range of patients.

## 4. Results

In our study, girls were frequently presented, 57%, versus 43% of boys. The age range was 11 to 21 years, with a mean of 15.8 ± 1.6. We found no statistical significance in age groups and side occurrence, as presented in Table 1. 

Age group 2 was the most frequent group, comprising more than half of the participants (56%), but the observed MRI parameters were not statistically significant in relation to age group, except the Caton–Deschamps index (*p* = 0.039), where the highest index value was noticed for age group 1 (1.13 ± 0.21) and the lowest for the age group 3 (1.03 ± 0.19). Even though we found no statistical significance for LTI (*p* = 0.612), TT-TG distance (*p* = 0.801), and Insall–Salvatti (*p* = 0.127) between age groups, the highest LTI was for age group 1 (15.75 ± 6.52), and the lowest was for the age group 3 (14.41 ± 5.89), while the TT-TG distance was highest for age group 1 (10.23 ± 5.01) and lowest for age group 2 (9.75 ± 4.34), and for Insall–Salvati, the highest index was for age group 1 (1.24 ± 0.21) and the lowest for age group 3 (1.14 ± 0.18). The data according to age groups are presented in Table 2.

Considering the patellar type in relation to gender and side affection, we found statistical significance related to gender (*p* = 0.035) but no statistical significance (*p* = 0.826) when considering side affection, as presented in Table 3. In age groups 1 and 2, females were more frequent (52.4% and 61.8%, respectively), while in the age group 3, males were more frequent (64.3%).

According to Wiberg morphological classification, the most frequent type was the second type, seen in about three quarters of tested individuals (74.6%), while the least frequent was the first patellar type, which was noticed in just above every tenth patient (10.9%) (Table 4).

The average LTI in different types of the patella was 14.79 ± 4.94 for the first patella type according to Wiberg, 16.29 ± 5.63 for the second, and 10.89 ± 6.72 for the third patellar type. The values are expressed in Table 4. According to our study, we found 50 participants with LTI < 11°, and that the patellar types correlate with LTI < 11°. In patellar type 1, 6 (28.6%) participants had LTI < 11°, compared to 28 (19.4%) in type 2 and 16 (57.1%) in type 3 (*p* < 0.001). For comparing LTI < 11° and patellar type, the Pearson chi-square test was used.

**Figure 3:**
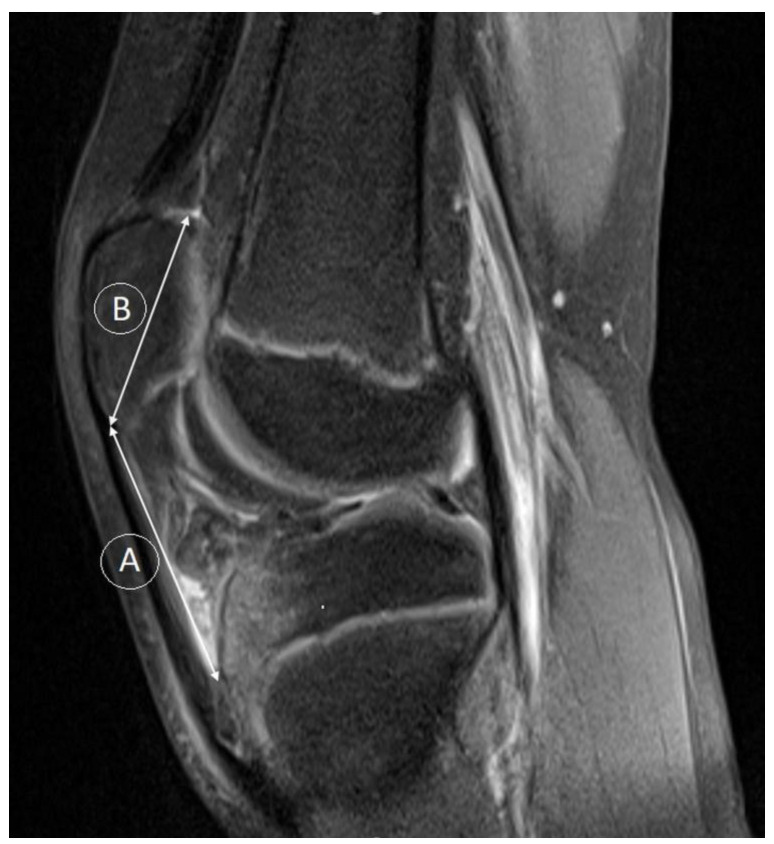
Patellar Insall–Salvati index (A:B). A—patellar tendon length, B—patellar length (described in the section Materials and Methods).

**Figure 4:**
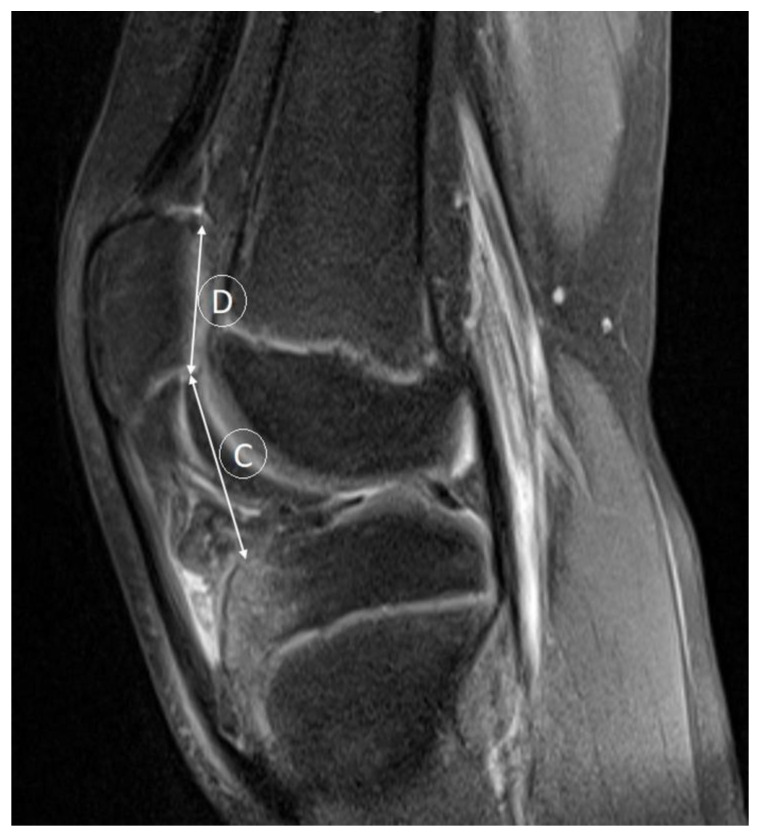
Patellar Caton–Deschamps index (C:D). C—distance from the inferior margin of the patellar articular surface, D—patellar tendon insertion length of the patellar articular surface (described in the section Materials and Methods).

Furthermore, there was a statistically significant association between patellar type groups and trochlear facet asymmetry (*p* < 0.001), Insall–Salvati index (Figure 3) (*p* = 0.001), and Caton–Deschamps index (Figure 4) (*p* = 0.018) (Table 4). Regarding TFA, the highest values were for patellar type 1 according to Wiberg (0.85 ± 0.10), and the lowest were for patellar type 3 according to Wiberg (0.46 ± 0.11). For the Insall–Salvati index, the highest values were for patellar type 3 according to Wiberg (1.33 ± 0.21), and the lowest were for patellar type 2 according to Wiberg (1.17 ± 0.20). For the Caton–Deschamps index, the highest values were for patellar type 3 according to Wiberg (1.17 ± 0.16), and the lowest were for patellar type 2 according to Wiberg (1.06 ± 0.19).

## 5. Discussion 

In our study, we have demonstrated that LTI, TFA, Insall–Salvati index, and Caton–Deschamps index significantly differed with regards to the patellar type according to Wiberg. Furthermore, only the Caton–Deschamps index, among the studied parameters, significantly differed with regards to the age groups of tested participants. 

The prediction of children’s injury rate using various morphological parameters of the knee has always been of interest and a debatable topic. In our study, it was shown that certain types of patella (according to Wiberg classification) might increase susceptibility for a knee injury, depending on the age of participants [14]. Furthermore, it was shown that the most common type of patella in knee injury (according to Wiberg) is type 2 in boys and girls type 3. 

Trochlear dysplasia (TD) is one of the leading causes of patellar instability [22]. The most commonly used MRI parameters for TD are LTI and TFA [23]. The LTI is commonly used to distinguish physiological from potentially dysplastic knees. LTI provides a quantitative description of dysplasia; it is reported that an LTI below 11° is associated with a 95% specificity of having patellar instability secondary to trochlear asymmetry [24], while a threshold of <0.4 or 40% is suggestive of trochlear dysplasia [25]. According to our study, the most vulnerable group of examined patients for TD was the one with the patellar type 2 due to the Wiberg classification. Carrillon et al. set the cut-off of 11 degrees for trochlear dysplasia, with very high sensitivity [15]. Stepanovich et al. explained that the existing growth potential affects the threshold, so the authors suggested the lower limit of 17 instead of 11 degrees [26]. Our results stress that the threshold for LTI might be at 11 degrees. Moreover, our findings indicate that certain patella types could be associated with increased susceptibility for TD in children after knee injury. Finally, we found that during skeletal maturation, LTI decreased, and TFA increased in a certain type of patella in knee injury in children. Moreover, the TFA ratio reported in our study was somewhat higher than that previously reported [27,28]. The possible explanation for our findings and the discrepancy compared to previous reports might be the fact that our study sample included sport-active adolescents. It was noticed that cartilage thickness at the patella does not strongly correlate with age [29], while physical activity might influence the thickness of articular cartilage [30]. 

The TT-TG distance value is also an essential parameter for evaluating patellofemoral disorders [31]. According to the literature, the LTI and TT-TG distance are used for diagnostics, treatment planning, and predicting future risk of re-dislocations [25]. Stepanovich et al. reported the correlation between TT-TG distance and patellar instability in patellar dislocation. They showed pathological values for TT-TG distance (over 20 mm) on their sample [26], which was not reflected in the results that we obtained. Our study found no statistical significance in TT-TG values in different patella types in different age groups. This might be explained by the differences in study design in regard to the observed groups. We observed participants with no injury history and with an absence of pathological clinical and X-ray findings, contrary to Stepanovich and co-workers. Additionally, the wide range of participants in different age groups in our study could be one of possible factors influencing the statistical outcome for the TT-TG parameter. Therefore, further study should include more homogenous age-group samples.

The position of the patella has an essential role in both knee stability and knee instability. The most frequently used measures in patella alta ratios are the Insall–Salvati, modified Insall–Salvati, and Caton–Deschamps indices [32,33]. We found statistical significance in the Caton–Deschamps index when considering the age groups of participants. According to different types of the patella (according to Wiberg), we found statistical significance of the Insall–Salvati index and the Caton–Deschamps index. The values of Insall–Salvati ratio in our study correspond with those of previous reports [34]. The same applies for the Caton–Deschamps index in our study [35]. Moreover, our study suggested that the Caton–Deschamps index is somehow more age-sensitive, because the knee cartilage changes described by this index were more detectable compared to the morphological changes described by the above-mentioned parameters. 

Furthermore, we showed that patella type 2 in boys and patella type 3 in girls (according to Wiberg) are the most prone to a knee injury in physically active children.

The comparisons of the present study results with those of the current literature are presented in Supplementary Material 1.

## 6. Conclusions

According to our study, the most frequent knee injury parameter in physically active pediatric patients is Wiberg patella type 2 in boys and type 3 in girls. Those patellar type morphological parameters, in association with high patella alta index, expressed as Insall–Salvati index and Caton–Deschamps index, could influence knee injury susceptibility in physically active children, especially in children over 14 years. 

The MRI morphometric parameters observed in our study might be factors of prediction of knee injury in physically active children. Moreover, it might be very useful in sports programs to improve the biomechanics of the knee in order to reduce the injury rate in sports-active children.

## Figures and Tables

**Table 1:** Knee injury prevalence by age groups.

		Age (Years)						*p-*Value
		11–14		15–17		18–21		
		*N* (%)	%	*N*	%	*N*	%	
Gender	male	22 (40.0)	40.0	48	44.4	13	43.3	0.863
	female	33 (60.0)	60.0	60	55.6	17	56.7	
Knee	Left	28 (50.9)	50.9	57	52.8	13	43.3	0.658
	right	27 (49.1)	49.1	51	47.2	17	56.7	

**Table 2:** Morphometric parameters by age groups.

Parameter Age Group (Years)		*N*	Mean ± SD	Median	*p-*Value
LTI	Group 1 (11–14)	55	15.75 ± 6.52	17.00	0.612
	Group 2 (15–17)	108	15.40 ± 5.79	15.85	
	Group 3 (18–21)	30	14.41 ± 5.89	15.50	
medial facet	Group 1 (11–14)	55	16.60 ± 2.78	16.60	0.357
	Group 2 (15–17)	108	17.03 ± 3.49	17.00	
	Group 3 (18–21)	30	17.65 ± 2.95	17.95	
lateral facet	Group 1 (11–14)	55	25.48 ± 3.72	25.00	0.861
	Group 2 (15–17)	108	25.52 ± 3.95	25.50	
	Group 3 (18–21)	30	25.92 ± 3.68	25.70	
trochlear facet asymmetry	Group 1 (11–14)	55	0.67 ± 0.14	0.69	0.707
	Group 2 (15–17)	108	0.68 ± 0.16	0.69	
	Group 3 (18–21)	30	0.69 ± 0.14	0.70	
TT-TG distance (mm)	Group 1 (11–14)	55	10.23 ± 5.01	10.10	0.801
	Group 2 (15–17)	108	9.75 ± 4.34	10.23	
	Group 3 (18–21)	30	9.81 ± 3.50	9.75	
Articular overlap	Group 1 (11–14)	55	12.70 ± 3.17	12.80	0.650
	Group 2 (15–17)	108	13.13 ± 2.94	13.10	
	Group 3 (18–21)	30	12.80 ± 2.58	12.55	
Insall–Salvati	Group 1 (11–14)	55	1.24 ± 0.21	1.23	0.127
	Group 2 (15–17)	108	1.20 ± 0.22	1.18	
	Group 3 (18–21)	30	1.14 ± 0.18	1.14	
MOD Insall–Salvati	Group 1 (11–14)	55	1.66 ± 0.23	1.64	0.062
	Group 2 (15–17)	108	1.57 ± 0.23	1.56	
	Group 3 (18–21)	30	1.56 ± 0.28	1.47	
Caton–Deschamps	Group 1 (11–14)	55	1.13 ± 0.21	1.10	0.039
	Group 2 (15–17)	108	1.07 ± 0.16	1.05	
	Group 3 (18–21)	30	1.03 ± 0.19	0.97	
Morphology ratio	Group 1 (11–14)	55	0.81 ± 0.08	0.82	0.463
	Group 2 (15–17)	108	0.82 ± 0.08	0.83	
	Group 3 (18–21)	30	0.82 ± 0.09	0.84	
Patellofemoral Contact Surface Ratio	Group 1 (11–14)	55	2.75 ± 0.73	2.55	0.979
	Group 2 (15–17)	108	2.77 ± 0.86	2.52	
	Group 3 (18–21)	30	2.77 ± 0.47	2.78	

**Table 3:** Knee injury prevalence by patellar type.

		Patellar Type						*p-*Value
		1		2		3		
		*N*	%	*N*	%	*N*	%	
Gender	male	10	47.6	55	38.2	18	64.3	0.035
	female	11	52.4	89	61.8	10	35.7	
Knee	Left	12	57.1	72	50.0	14	50.0	0.826
	right	9	42.9	72	50.0	14	50.0	

**Table 4:** Morphometric parameters by patellar type (Wiberg classification).

Parameter Patellar Type		*N*	Mean (± SD)	Median	*p-*Value
LTI	1	21	14.79 ± 4.94	16.10	<0.001
	2	144	16.29 ± 5.63	16.80	
	3	28	10.89 ± 6.72	9.25	
medial facet	1	21	19.17 ± 2.58	19.50	<0.001
	2	144	17.33 ± 2.95	17.40	
	3	28	13.69 ± 2.66	13.70	
lateral facet	1	21	22.59 ± 2.95	22.20	<0.001
	2	144	25.13 ± 3.25	25.25	
	3	28	30.09 ± 3.54	30.00	
trochlear facet asymmetry	1	21	0.85 ± 0.10	0.87	<0.001
	2	144	0.69 ± 0.11	0.70	
	3	28	0.46 ± 0.11	0.46	
TT-TG distance (mm)	1	21	9.20 ± 3.98	10.20	0.086
	2	144	10.29 ± 4.40	10.05	
	3	28	8.40 ± 4.51	7.77	
Articular overlap	1	21	13.15 ± 3.35	13.10	0.952
	2	144	12.93 ± 2.70	13.00	
	3	28	12.94 ± 3.85	13.15	
Insall–Salvati	1	21	1.22 ± 0.23	1.18	0.001
	2	144	1.17 ± 0.20	1.15	
	3	28	1.33 ± 0.21	1.36	
MOD Insall–Salvati	1	21	1.60 ± 0.19	1.59	0.834
	2	144	1.59 ± 0.25	1.56	
	3	28	1.62 ± 0.22	1.61	
Caton–Deschamps	1	21	1.07 ± 0.13	1.04	0.018
	2	144	1.06 ± 0.19	1.03	
	3	28	1.17 ± 0.16	1.14	
Morphology ratio	1	21	0.82 ± 0.07	0.82	0.059
	2	144	0.81 ± 0.08	0.83	
	3	28	0.85 ± 0.06	0.85	
PF Contact Surface Ratio	1	21	2.76 ± 0.87	2.48	0.308
	2	144	2.73 ± 0.67	2.56	
	3	28	2.97 ± 1.10	2.53	

## Data Availability

All the data are available from the corresponding author upon reasonable request.

## Supplementary Materials

The following supporting information can be downloaded at: https://www.mdpi.com/article/10.3390/medicina59020213/s1, Table S1: material 1.
