# Supplementary material for: Morphometric Parameters and MRI Morphological Changes of the Knee and Patella in Physically Active Adolescents
Source: Medicina (Kaunas). 2023 Jan 22;59(2):213. doi: 10.3390/medicina59020213 (PMC9965593; doi:10.3390/medicina59020213)
Supplement: Supplementary file 1 [file medicina-59-00213-s001.zip › medicina-2108351-supplementary.pdf]

Supplementary material S1

Study findings	Current literature
LTI threshold might be at 11 degrees	Carrillon et al., set the cut-off of 11 degrees for trochlear dysplasia, with very high sensitivity [12]<br>Stepanovich et al., suggested the lower limit of 17 instead of 11 degrees [21]
TT-TG distance for group 1 (10.23±5.01 mm), group 2 (9.75±4.34 mm), group 3 (9.81±3.50 mm)	Stepanovich et al., proposed pathological values for TT-TG distance over 20 mm [21]
TFA ratio was for group 1 (0.67±0.14), group 2 (0.68±0.16), group 3 (0.69±0.14)	Shen et al., stressed out that for measurement based on cartilage landmark TFA ratio was 0.4±0.1 [24]<br>Kim and Parikh pointed that TFA ratio bellow 0.4 is an indicator for trochlear dysplasia (100% sensitivity and 96% specificity) [25]
Insall-Salvati ratio for group 1 (1.22±0.23), group 2 (1.17±0.20), group 3 (1.33±0.21)	Kurowecki et al., stated that mean value of Insall-Salvati was 1.22 [31]
Caton-Deschamps index for group 1 (1.13± 0.21), group 2 (1.07± 0.16), group 3 (1.03± 0.19)	Thévenin-Lemoine et al., stated that the mean Caton-Deschamps index was 1.06 ± 0.21 [32]
